# Supplementary figures and images for: Cellular Variability of RpoS Expression Underlies Subpopulation Activation of an Integrative and Conjugative Element
Source: PLoS Genet. 2012 Jul 12;8(7):e1002818. doi: 10.1371/journal.pgen.1002818 (PMC3395598; doi:10.1371/journal.pgen.1002818)

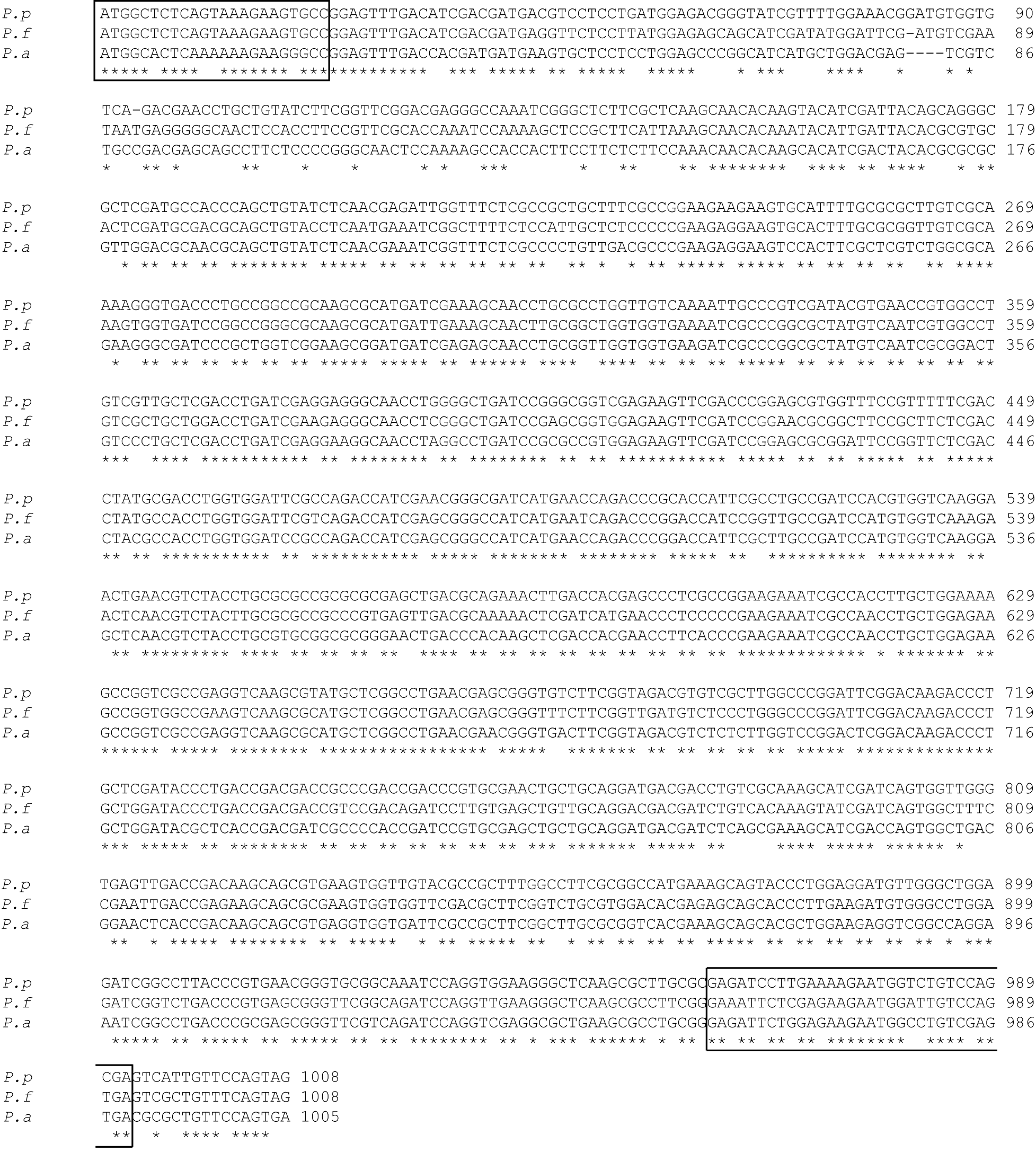

Supplement: Figure S1 — Alignment of rpoS genes from Pseudomonas putida (P. p.), P. fluorescens (P. f.) and P. aeruginosa (P. a.). Rectangular boxes represent the region chosen to design primers for the amplification of rpoS from strain B13. Inosine was used in the oligonucleotides at non-conserved positions. Genbank numbers: P. putida KT2440, NC_002947.3; P. fluorescens Pf-5, NC_004129.6; P.aeruginosa PAO1, NC_002516.2. (TIF) [file pgen.1002818.s001.tif]

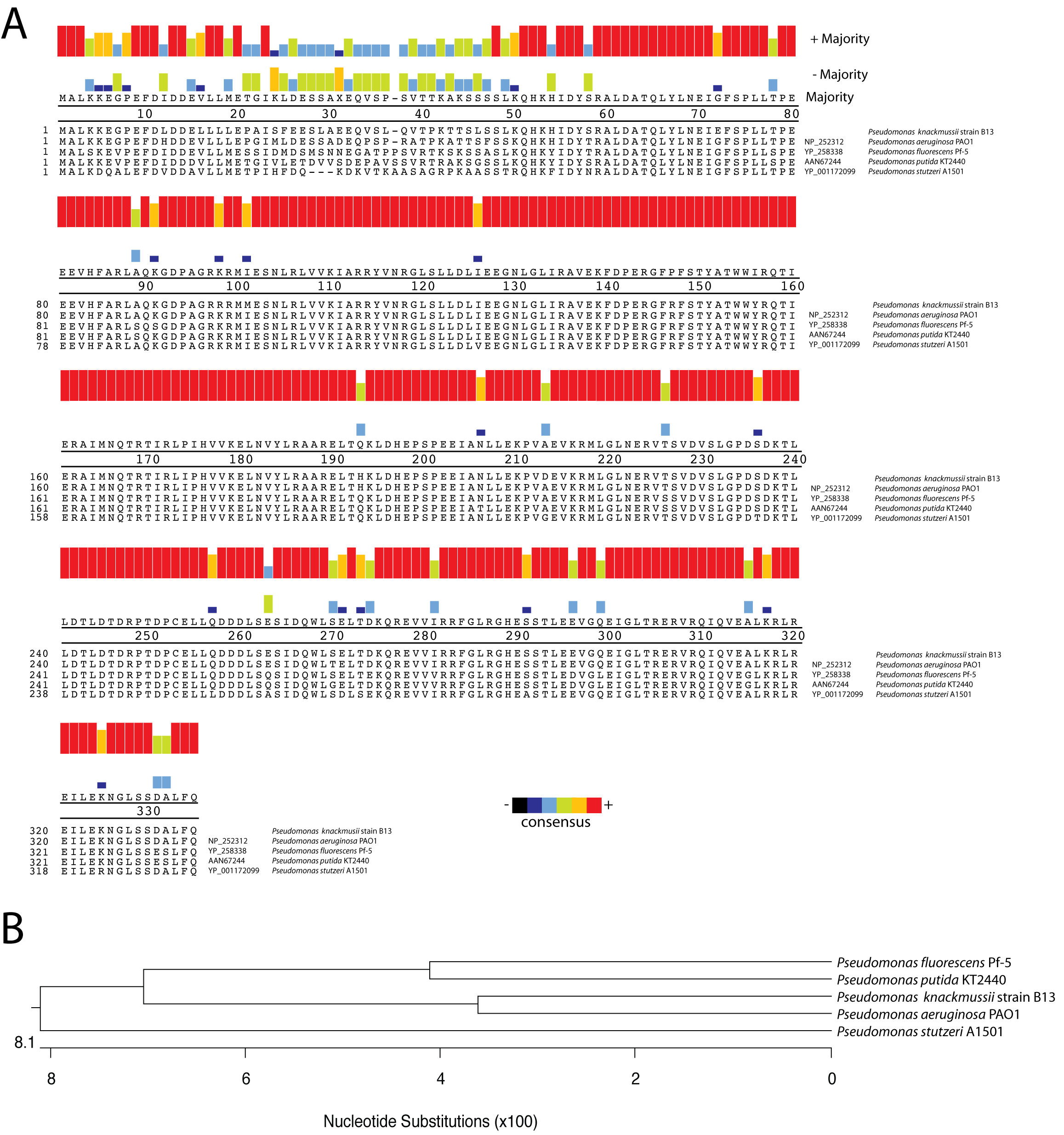

Supplement: Figure S2 — Comparison of the predicted RpoS amino acid sequence from strain B13 and orthologues from four other Pseudomonas strains. (A) MegAlign alignment (DNAStar Lasergene package v.8) and indication of consensus per position. (B) Dendrogram (Clustal 2.0.12, http://www.ebi.ac.uk) showing the closest neighbourhood clustering of the strain B13 rpoS gene. (TIF) [file pgen.1002818.s002.tif]

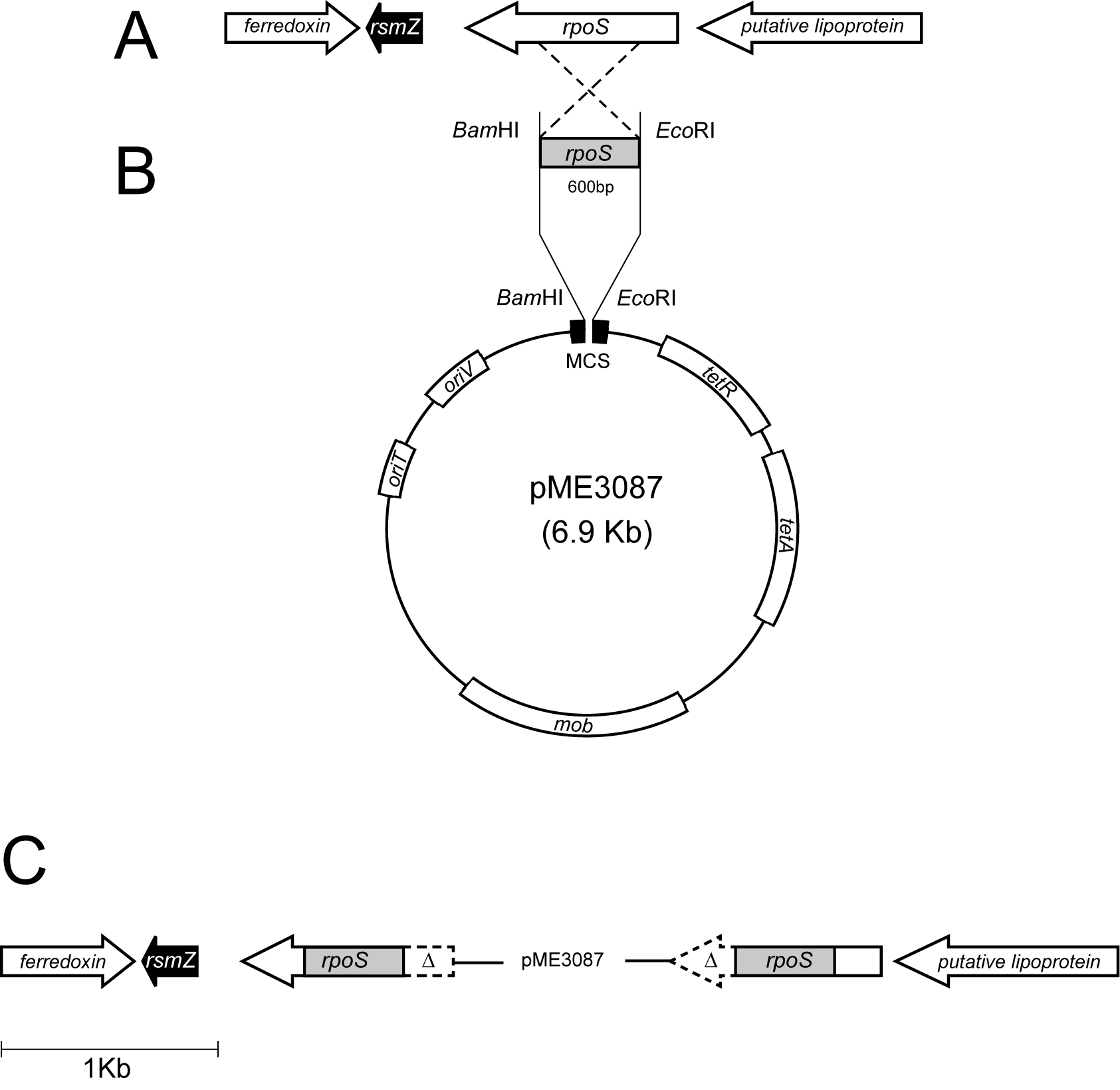

Supplement: Figure S3 — Strategy for inactivating rpoS in strain B13 by a single recombination event. (A) rpoS gene region. (B) Amplification of a 600-bp internal rpoS B13 fragment by PCR whilst creating BamHI and EcoRI restriction sites. Insertion of the rpoS B13 fragment into the suicide vector pME3087. (C) Genetic structure produced by single homologous recombination and inactivation of rpoS on the B13 chromosome. (TIF) [file pgen.1002818.s003.tif]

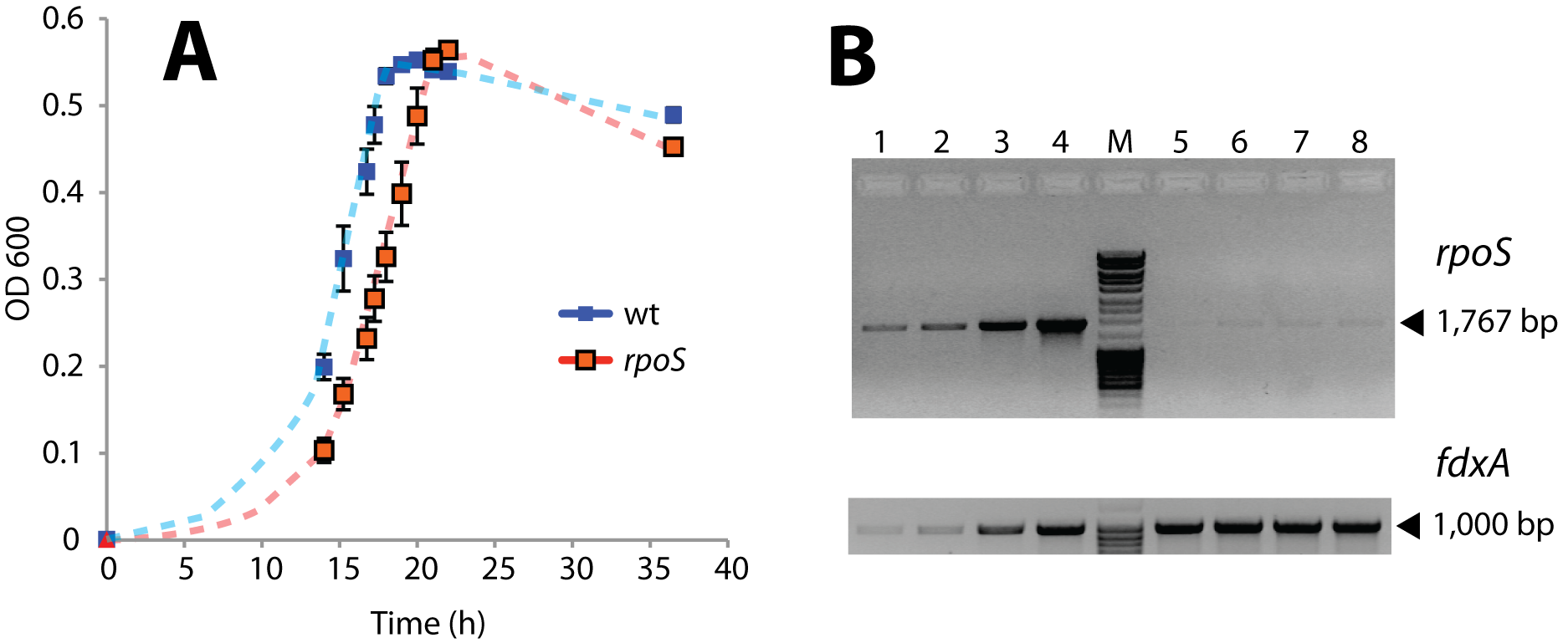

Supplement: Figure S4 — Growth of P. knackmussii B13-78 wild-type and B13-2671 (rpoS) in MM with 5 mM 3CBA. Data points are the average from three independent biological replicates ± one calculated standard deviation. Maximal specific growth rates in exponential phase for B13-78 were 0.22±0.01 versus 0.26±0.01 h−1 for B13-2671 (rpoS). Note that growth medium for B13-2671 included Tc to select for the rpoS-pME3087 allele. (B) Semi-quantification of the presence of rpoS revertants in B13-2671 (rpoS) cultures by PCR. 25 ng of genomic DNAs isolated from B13-2671 culture with Tc at 24 h (lane 5), 48 h (lane 6), 72 h (lane 7), or 96 h (lane 8) were used as templates. A serially diluted B13-78 (wild-type) DNA was used as control: lane 1, 0.25 ng; lane 2, 0.5 ng; lane 3, 2.5 ng; lane 4, 25 ng. Intact rpoS (upper panel) and fdxA (lower panel, as an internal control) alleles were amplified using primer pairs 090206+090902 and 110524+110525, respectively. Lane M, molecular mass marker (MassRuler DNA Ladder, Fermentas). The positions and sizes of the expected PCR fragments are indictaed. Note that some reversion of rpoS-pME3087 to wild-type rpoS must occur (lane 7–9) but at less than 1% in the population (lane 1). (TIF) [file pgen.1002818.s004.tif]

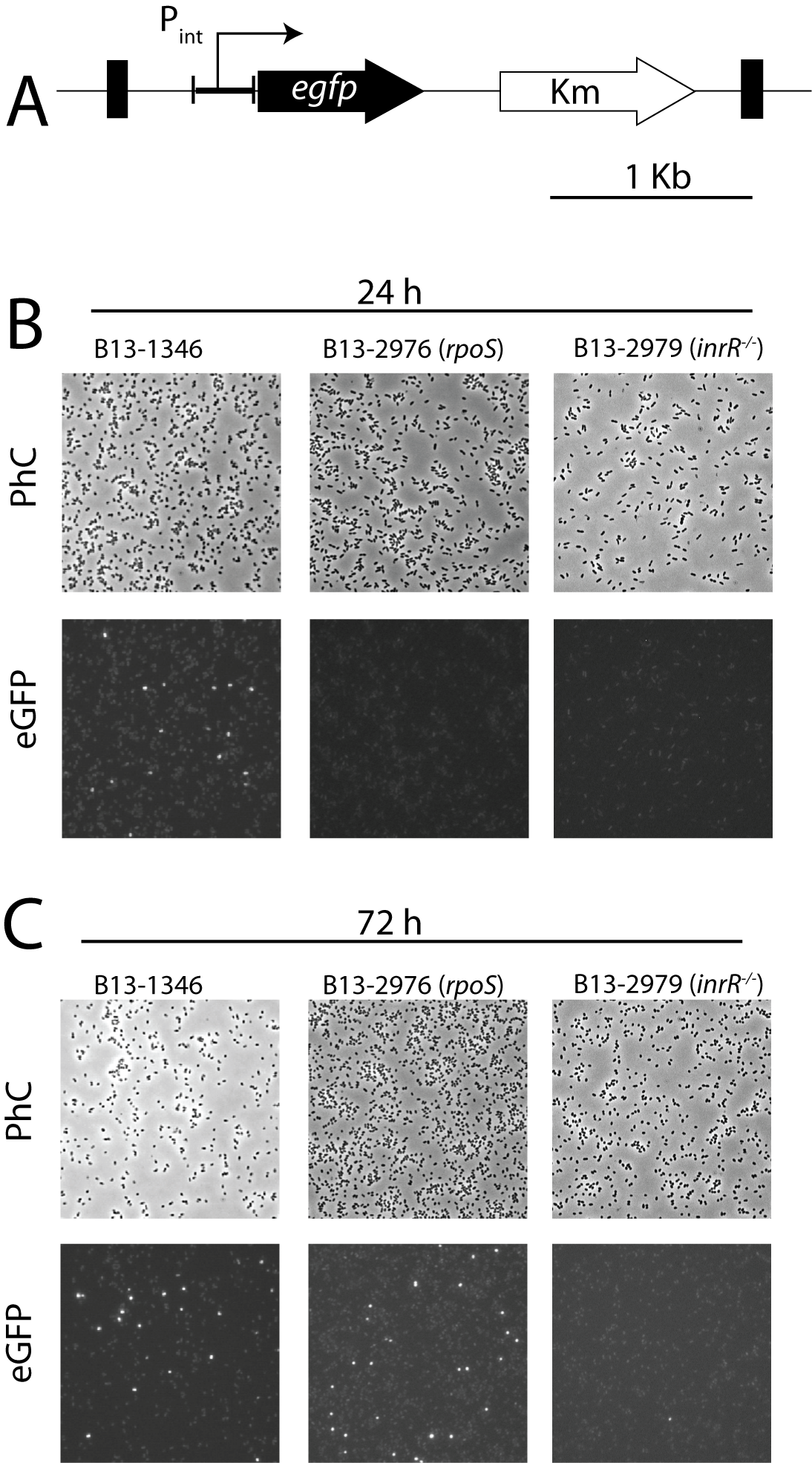

Supplement: Figure S5 — Comparison of effects caused by rpoS or double inrR disruption on expression of a Pint-egfp fusion in P. knackmussii. (A) Relevant construction details of the mini-Tn construct delivering the single copy Pint-egfp fusion. (B) Micrographs showing the subpopulation of cells expressing eGFP from Pint amidst a large number of silent cells for B13-1346 (wild-type), B13-2976 (rpoS) or B13-2979 (inrR−/−) cultured on 3CBA after 24 h into stationary phase. (C) As B, but after 72 h in stationary phase. Shown are phase-contrast micrographs at 1,000× magnification and corresponding epifluorescence images. For quantification, see Table 3. (TIF) [file pgen.1002818.s005.tif]

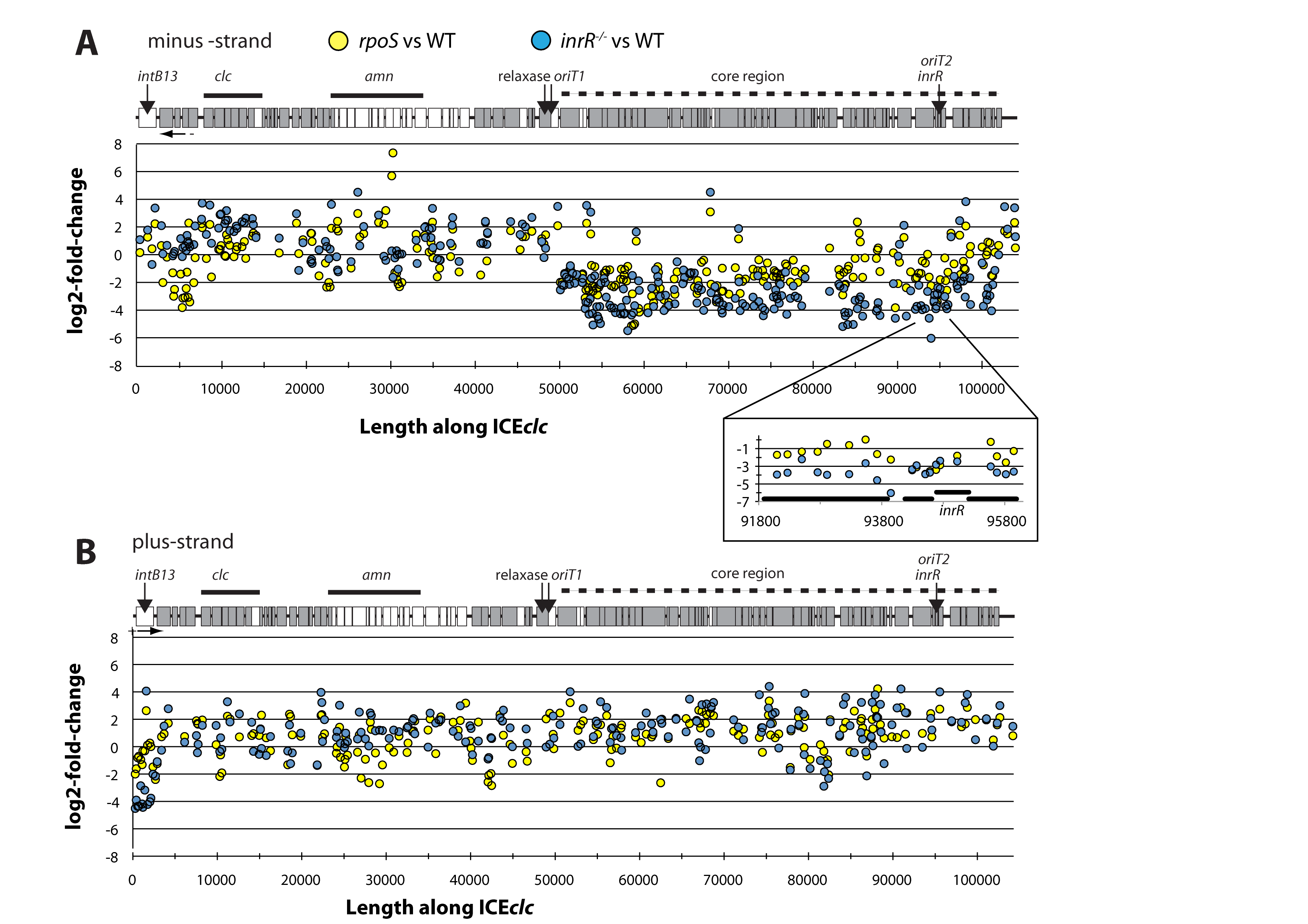

Supplement: Figure S6 — ICEclc gene expression compared among P. knackmussii B13-78 (wild-type), B13-2201 (inrR−/−) and B13-2671 (rpoS). A) Log2 fold-change in negative-strand probe signals on an ICEclc micro-array. Inset shows detail around inrR-operon. B) Positive-strand probe signals. Open reading frames of ICEclc plotted along its length; white boxes: genes oriented on the positive strand, grey boxes: negative strand. Known ICEclc functional genes or regions indicated by name for reference. (TIF) [file pgen.1002818.s006.tif]

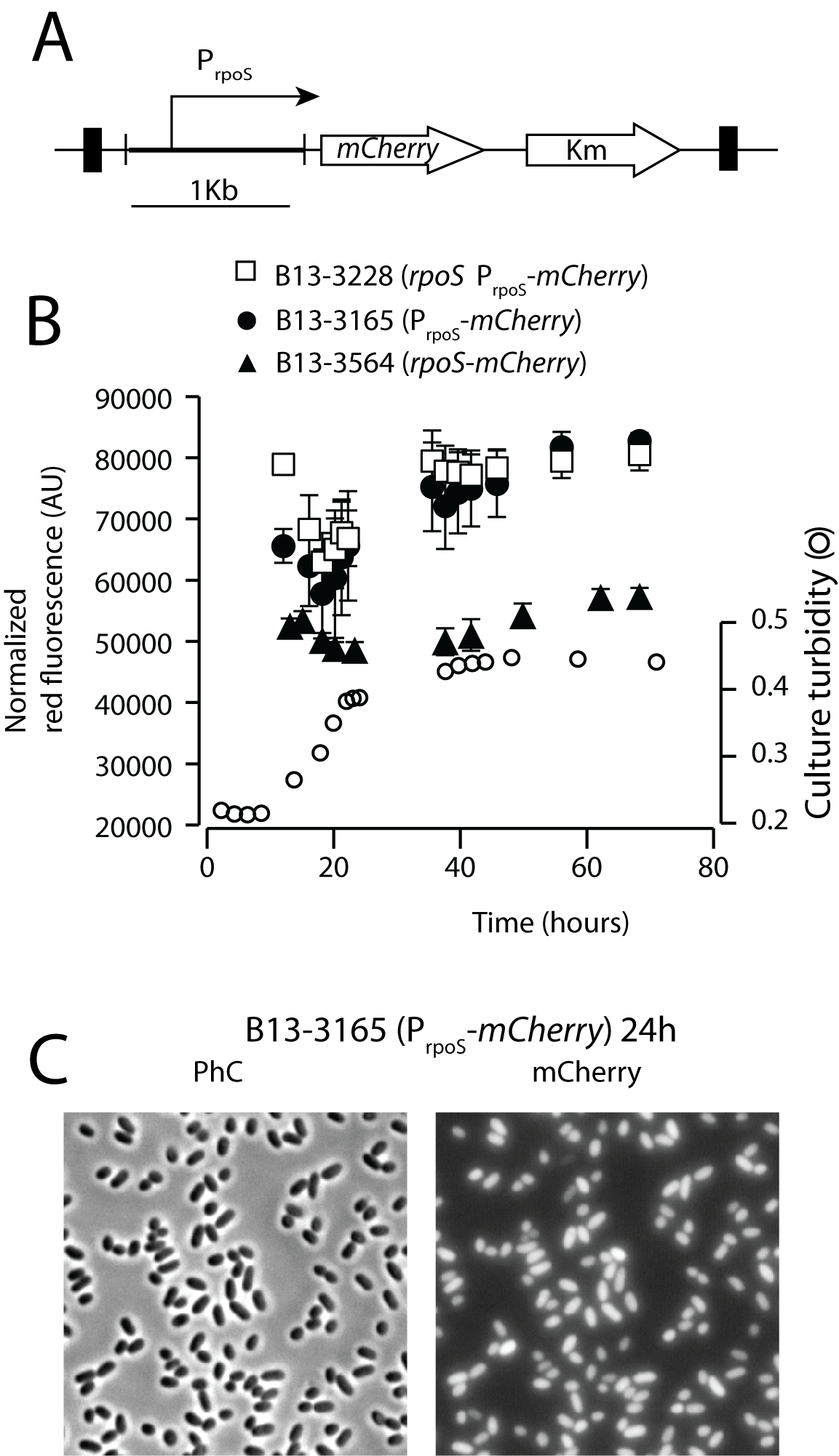

Supplement: Figure S7 — Growth phase dependent expression from the rpoS promoter in P. knackmussii. (A) Relevant construction details of the mini-Tn construct used to place a single copy PrpoS-mCherry transcriptional fusion in the B13 genome. (B) Culture-density normalized mCherry fluorescence as a function of culture density (open circles) and incubation time in B13-3165 (wild-type) B13-3228 (rpoS), or B13-3654 (rpoS-mCherry). (C) Corresponding phase contrast (PhC) and epifluorescence micrographs of B13-3165 cells 24 h into stationary phase. Note how expression from PrpoS is RpoS independent and how expression of RpoS-mCherry from PrpoS is detectable slightly later than that of mCherry alone, suggesting post-transcriptional effects. (TIF) [file pgen.1002818.s007.tif]

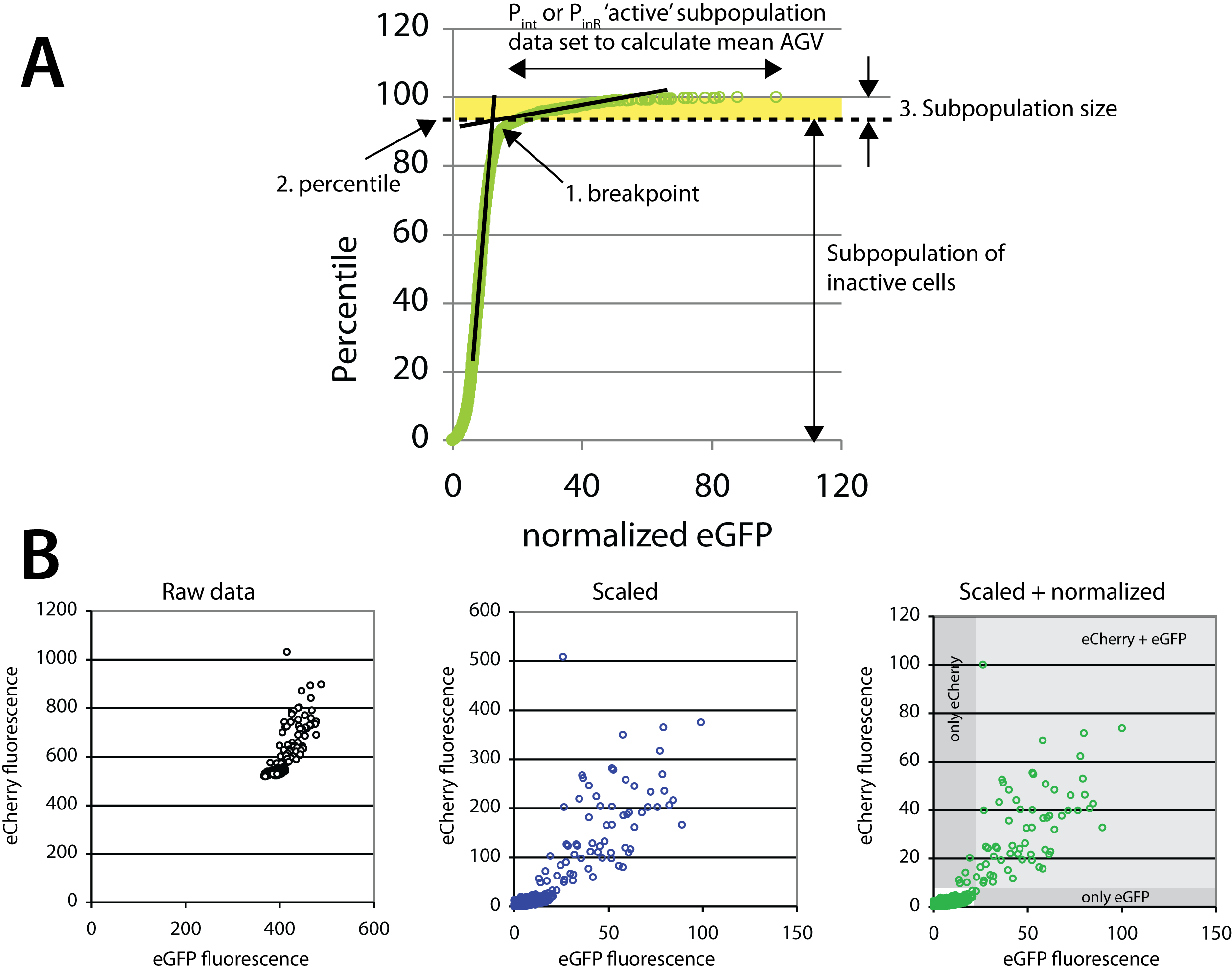

Supplement: Figure S8 — Calculation of the subpopulation (size and mean reporter fluorescence expression) of B13-cells expressing Pint or PinR above threshold and representative for activating the ICEclc element. (A) Finding the breakpoint between the larger non-active subpopulation of cells and the smaller ICEclc-active subpopulation of cells on a cumulative distribution curve of reporter fluorescence values from Pint or PinR. (B) Scaling and normalizing of eCherry and eGFP expression for noise calculations. Only cells falling in the grey zones (i.e., those with reporter expression values above the threshold defined in [A]) are considered for noise calculation. (TIF) [file pgen.1002818.s008.tif]
