# Supplementary material for: Cellular Variability of RpoS Expression Underlies Subpopulation Activation of an Integrative and Conjugative Element
Source: PLoS Genet. 2012 Jul 12;8(7):e1002818. doi: 10.1371/journal.pgen.1002818 (PMC3395598; doi:10.1371/journal.pgen.1002818)
Supplement: Table S1 — Transfer frequencies of ICEclc from P. knackmussii strain B13, the inrR deletion and the rpoS deletion mutants to P. putida UWC1 as recipient. (DOC) [file pgen.1002818.s009.doc]

**Table S1.** Transfer frequencies of ICE*clc* from *P. knackmussii* strain B13, the *inrR* deletion and the *rpoS* deletion mutants to *P. putida* UWC1 as recipient.

| **Mating time (h)** | *Pseudomonas knackmussii* B13 | | | | | | | |
| --- | --- | --- | --- | --- | --- | --- | --- | --- |
|  | **A) Wild-type** |  | **B) *rpoS*** |  | **C) *rpoS* miniTn(*rpoS*)** |  | **D) *rpoS*, *inrR^-/-^*** |  |
|  | Transfer^a^ | T-test |  | T-test |  | T-test |  |  |
| 24 | 1.1 ± 0.2·10^-2^ | AB, AD | 3.7 ± 1.1·10^-5^ | BC | 1.1 ± 0.4·10^-2^ | CD | 3.2 ± 0.9·10^-5^ |  |
| 48 | 2.7 ± 0.3·10^-2^ | AB, AD | 3.8 ± 0.8·10^-5^ | BC | 1.8 ± 0.2·10^-2^ | CD | 2.3 ± 0.3·10^-5^ |  |
| 72 | 6.1 ± 0.4·10^-2^ | AB, AD | 9.3 ± 1.3·10^-3^ | BC, BD | 7.0 ± 1.6·10^-2^ | CD | 1.2 ± 0.3·10^-4^ |  |
| 96 | 1.1 ± 0.3·10^-1^ | AB, AD | 4.7 ± 0.3·10^-2^ | BC, BD | 1.2 ± 0.2·10^-1^ | CD | 8.9 ± 0.7·10^-4^ |  |

a) Transfer frequencies as colony forming units (CFU) of *P. putida* transconjugants per CFU donor

b) Significance (P<0.005) in pair-wise homoscedastic T-test.
