# Supplementary material for: Cellular Variability of RpoS Expression Underlies Subpopulation Activation of an Integrative and Conjugative Element
Source: PLoS Genet. 2012 Jul 12;8(7):e1002818. doi: 10.1371/journal.pgen.1002818 (PMC3395598; doi:10.1371/journal.pgen.1002818)
Supplement: Table S2 — Transfer frequencies of ICEclc from P. knackmussii strain B13 and the rpoS + strain to P. putida UWC1 as recipient. (DOC) [file pgen.1002818.s010.doc]

**Table S2.** Transfer frequencies of ICE*clc* from *P. knackmussii* strain B13 and the *rpoS*^+^ strain to *P. putida* UWC1 as recipient.

| Mating time | Transfer frequency^a^ | | P-value^b^ |
| --- | --- | --- | --- |
|  | B13 wt | B13-3201 (*rpoS*^+^) |  |
| 48h | 1.3 ± 0.1 × 10^-2^ | 2.4 ± 0.9 × 10^-2^ | 0.097 |

a) Transfer frequencies as colony forming units (CFU) of *P. putida* transconjugants per CFU donor.

b) P-value calculated from pair-wise homoscedastic t-test.
