# Supplementary material for: Cellular Variability of RpoS Expression Underlies Subpopulation Activation of an Integrative and Conjugative Element
Source: PLoS Genet. 2012 Jul 12;8(7):e1002818. doi: 10.1371/journal.pgen.1002818 (PMC3395598; doi:10.1371/journal.pgen.1002818)
Supplement: Table S3 — Effect of subpopulation size on noise calculation from two identical Pint-copies in different places on the chromosome of P. knackmussii derivatives. (DOC) [file pgen.1002818.s011.doc]

**Table S3.** Effect of subpopulation size^a^ on noise calculation from two identical P_int_-copies in different places on the chromosome of *P. knackmussii* derivatives.

| Strain  Noise | B13-2717  P*_int_*-*egfp*, P*_int_*-*echerry* | B13-3201 (*rpoS^+^*)  P*_int_*-*egfp*, P*_int_*-*echerry* | P-value |
| --- | --- | --- | --- |
| Intrinsic noise | 1.17 ± 0.13^b^ | 0.57 ± 0.10 | 0.003 |
| Extrinsic noise | 0.31 ± 0.25 | 0.47 ± 0.03 | 0.391 |
| Total noise | 1.22 ± 0.17 | 0.74 ± 0.09 | 0.013 |

a) The subpopulations sizes of ICE*clc* active cells (expressing P_int_ above threshold) in both strains were adjusted to 6% by resampling data sets of Table 4 in 1000 bootstrappings.

b) Noise values are the averages across all subsampled values ± one *SD*. P-value calculated from pair-wise homoscedastic t-test
