# Supplementary material for: Cellular Variability of RpoS Expression Underlies Subpopulation Activation of an Integrative and Conjugative Element
Source: PLoS Genet. 2012 Jul 12;8(7):e1002818. doi: 10.1371/journal.pgen.1002818 (PMC3395598; doi:10.1371/journal.pgen.1002818)
Supplement: Table S4 — Primers used in this study. (DOC) [file pgen.1002818.s012.doc]

**Table S4.** Primers used in this study.

| Number | Sequence 5’-3’^a^ | Location |
| --- | --- | --- |
| 061001 | ATGGCICTCAIIAAAGAAGIGCC | From *rpoS* alignment |
| 061002 | TCICTIGACAIICCATTCTTITC | From *rpoS* alignment |
| 080303 | TTTTGAATTCCCCAGCTGTATCTCAACGAAATC | *rpoS* of B13 (*Eco*RI site) |
| 080304 | TTTTTGGATCCATCGGGCGGTCATCGGT | *rpoS* of B13 (*Bam*HI site) |
| 100104 | TTTTTTGCGGCCGCCGTTTCGTCCCGCTGCTCAACGG | *P. aeruginosa nlpD* (*Not*I site) |
| 100103 | TTTTTTGAATTCGGCGGGCTCCAGCAACAGCAG | *rpoS* of B13 (*Eco*RI site) |
| 060605 | TTTTTTGAATTCGCGCAATCACCGATCGCGCAT | P*_inR_*-*orf95213*-*inrR* on ICE*clc* (*Eco*RI site) |
| 080502 | TTTTTACTAGTGACCTTCTGCGCAAGAGCTGA | P*_inR_*-*orf95213*-*inrR* on ICE*clc* (*Spe*I site) |
| 090206 | ATTGCCGAGATGGGCTCC | *fdxA* of B13 |
| 090902 | TTTTTTGAATTCCCCGAGAACATGCAGGAGTTC | P*_rpoS_*-*rpoS* of B13 (*Eco*RI site) |
| 091206 | TTTTTTGCATGCCGACACCCTGTATTCCATTGCC | P*_rpoS_*-*rpoS* of B13 (*Sph*I site) |
| 101001 | GGGGGAATTCATGCATCGTTTCGTCCCTCTACTCAATGG | P*_rpoS_*-*rpoS* of B13 (*Eco*RI and *Nsi*I site) |
| 101002 | GGGGAAGCTTCTGGAACAGGGCGTCGCTC | P*_rpoS_*-*rpoS* without stop codon of B13 (*Hin*dIII site) |
| 101003 | GGGGAAGCTT*CCGGAAAATTCGAACGTTACGCGTCACCGGTCGGCCACC*GTTTCCAAGGGCGAGGAGG | *mcherry* without start codon of pMQ64-mcherry (*Hin*dIII site) |
| 101004 | GGGGACTAGTTTATTTGTACAGCTCATCCATGCC | *mcherry* of pMQ64-mcherry (*Spe*I site) |
| 110524 | GGGGTCTAGACAGGTGATCAGATGAAAGAGC | Downstream region of *rpoS* of B13 (*Xba*I site) |
| 110525 | GGGGCTCGAGTGGAAACCACCAGCCTGC | Downstream region of *rpoS* of B13 (*Xho*I site) |

a) Restriction sites are underlined. Sequence encoding linker peptides are indicated in italic.
